# Supplementary material for: Cyprinid herpesvirus 3 Evolves In Vitro through an Assemblage of Haplotypes that Alternatively Become Dominant or Under-Represented
Source: Viruses. 2019 Aug 15;11(8):754. doi: 10.3390/v11080754 (PMC6723609; doi:10.3390/v11080754)
Supplement: Supplementary file 1 [file viruses-11-00754-s001.pdf]

**Table S 1.** Nature and position of variants between P78 and P0.

| | | | | | | P78-1c | | P78-2c | | P78-1s | | P78-2s | |
|---|---|---|---|---|---|---|---|---|---|---|---|---|---|
| **#** | **Position on KHV-J Reference** | **Reference Sequence** | **Alternate Sequence** | **Type of Variation** | **Predicted ORF** | **Allele Freq – Reference** | **Allele Freq – Alternate** | **Allele Freq – Reference** | **Allele Freq – Alternate** | **Allele Freq – Reference** | **Allele Freq – Alternate** | **Allele Freq – Reference** | **Allele Freq – Alternate** |
| 1 | 17848 | C | G | missense variant | KHVJ011 | 0.80 | 0.20 | 0.75 | 0.25 | 0.76 | 0.24 | 0.72 | 0.28 |
| 2 | 17849 | G | A | missense variant | KHVJ011 | 0.80 | 0.20 | 0.74 | 0.26 | 0.78 | 0.22 | 0.73 | 0.27 |
| 3 | 17850 | C | G | synonymous variant | KHVJ011 | 0.80 | 0.20 | 0.74 | 0.26 | 0.79 | 0.21 | 0.74 | 0.26 |
| 4 | 26023 | G | A | missense variant | KHVJ019 | 0.56 | 0.44 | 0.60 | 0.40 | 0.55 | 0.45 | 0.54 | 0.46 |
| 5 | 34507 | T | TC | frameshift variant | KHVJ024 | 0.62 | 0.38 | 0.65 | 0.35 | 0.63 | 0.37 | 0.62 | 0.38 |
| 6 | 83069 | C | CCGAAGCAGCCGAAGCAGCCGAAGCAGCCGAAGCAGA | inframe insertion | KHVJ055 | 0.78 | 0.22 | 0.85 | 0.15 | 0.72 | 0.28 | 0.79 | 0.21 |
| 7 | 124200 | AGGGG | A | intergenic variant | KHVJ075-KHVJ076 | 0.38 | 0.63 | 0.40 | 0.60 | 0.62 | 0.38 | 0.31 | 0.69 |
| 8 | 138300 | C | A | missense variant | KHVJ082 | 0.00 | 1.00 | 0.00 | 1.00 | 0.00 | 1.00 | 0.00 | 1.00 |
| 9 | 154881 | G | T | synonymous variant | KHVJ093 | 0.82 | 0.18 | 0.85 | 0.15 | 0.86 | 0.14 | 0.83 | 0.17 |
| 10 | 157768 | C | A | missense variant | KHVJ095 | 0.10 | 0.90 | 0.08 | 0.92 | 0.11 | 0.89 | 0.09 | 0.91 |
| 11 | 187878 | GTCT | G | inframe deletion | KHVJ111 | 0.87 | 0.13 | 0.91 | 0.09 | 0.87 | 0.13 | 0.90 | 0.10 |

| | | | | | | | | | | | | | |
|---|---|---|---|---|---|---|---|---|---|---|---|---|---|
| 12 | 193352 | G | A | missense variant | KHVJ113/ KHVJ114 | 0.66 | 0.34 | 0.63 | 0.37 | 0.60 | 0.40 | 0.65 | 0.35 |
| 13 | 209653 | A | T | missense variant | KHVJ125 | 0.65 | 0.35 | 0.62 | 0.38 | 0.56 | 0.44 | 0.55 | 0.45 |
| 14 | 223325 | A | C | missense variant | KHVJ138 | 0.00 | 1.00 | 0.00 | 1.00 | 0.00 | 1.00 | 0.00 | 1.00 |
| 15 | 256979 | C | T | missense variant | KHVJ159 | 0.00 | 1.00 | 0.00 | 1.00 | 0.00 | 1.00 | 0.00 | 1.00 |
| 16 | 257090 | C | T | missense variant | KHVJ159 | 0.00 | 1.00 | 0.00 | 1.00 | 0.00 | 1.00 | 0.00 | 1.00 |
| 17 | 258111 | C | T | intergenic variant | KHVJ159- KHVJ160 | 0.26 | 0.74 | 0.27 | 0.73 | 0.32 | 0.68 | 0.36 | 0.64 |
| 18 | 259695 | CG | C | frameshift variant | KHVJ160 | 0.69 | 0.31 | 0.73 | 0.27 | 0.67 | 0.33 | 0.67 | 0.33 |
| 19 | 269057 | TTCC | T | inframe deletion | KHVJ166 | 0.66 | 0.34 | 0.59 | 0.41 | 0.61 | 0.39 | 0.45 | 0.55 |
| 20 | 270260 | AGAGGC AGAG | A | inframe deletion | KHVJ166/ KHVJ167 | 0.73 | 0.27 | 0.71 | 0.29 | 0.71 | 0.29 | 0.72 | 0.28 |
| 21 | 272753 | T | TGGTGGA AGGTTGA ACATGGT GGACAC. TGGTGGA AGGTTGA ACATGGT GGACAC GGTGGA AGGTTGA ACATGGT GGACAC | frameshift variant | KHVJ169 | 0.59 | 0.41 | 0.28 | 0.72 | 0.50 | 0.50 | 0.38 | 0.62 |

All variations located in the terminal repeats appear only once. Predicted ORFs were deduced from the genome annotation of KHV-J (AP008984). Missense variant indicates a nucleotide substitution that modifies the amino acid; synonymous variant denotes a nucleotide substitution that does not modify the amino acid; frameshift variant indicates

an insertion or a deletion that changes the reading frame; intergenic variant points to a mutation located between two predicted open reading frames. When more than two variants co-occur at a single position, only the frequency of the most abundant alternate (from all replicates) is indicated. Only variations <100 bp were listed.

**Table S 2.** Nature and position of variants between P99 and P0.

| | | | | | | P99-1c | | P99-2c | |
|---|---|---|---|---|---|---|---|---|---|
| # | Position on KHV-J Reference | Reference Sequence | Alternate Sequence | Type of Variation | Predicted ORF | Allele Freq – Reference | Allele Freq – Alternate | Allele Freq – Reference | Allele Freq – Alternate |
| 1 | 7090 | C | CA | intergenic variant | KHVJ04-KHVJ05 | 0.00 | 1.00 | 0.00 | 1.00 |
| 2 | 7300 | C | A | missense variant | KHVJ05 | 0.00 | 1.00 | 0.00 | 1.00 |
| 3 | 8635 | GAGCACC | G | inframe deletion | KHVJ06 | 0.00 | 1.00 | 0.00 | 1.00 |
| 4 | 8926 | G | T | missense variant | KHVJ06 | 0.00 | 1.00 | 0.00 | 1.00 |
| 5 | 8963 | GC | G | frameshift variant | KHVJ06 | 0.00 | 1.00 | 0.00 | 1.00 |
| 6 | 9201 | G | A | missense variant | KHVJ06 | 0.00 | 1.00 | 0.00 | 1.00 |
| 7 | 9709 | T | C | missense variant | KHVJ06 | 0.00 | 1.00 | 0.00 | 1.00 |
| 8 | 11333 | A | ATC | intergenic variant | KHVJ06-KHVJ07 | 0.00 | 1.00 | 0.03 | 0.97 |
| 9 | 22308 | AAATTAGGGCTAG | A | intergenic variant | KHVJ015-KHVJ016 | 0.84 | 0.16 | 0.00 | 1.00 |
| 10 | 23622 | T | C | synonymous variant | KHVJ017 | 0.88 | 0.12 | 0.82 | 0.18 |
| 11 | 24969 | C | CCAA | inframe insertion | KHVJ018 | 0.88 | 0.12 | 0.90 | 0.10 |
| 12 | 31414 | ACT | A | frameshift variant | KHVJ023 | 0.66 | 0.34 | 0.86 | 0.14 |

| | | | | | | | | | |
|---|---|---|---|---|---|---|---|---|---|
| 13 | 37042 | C | CTGT | inframe insertion | KHVJ026 | 0.90 | 0.10 | 0.70 | 0.30 |
| 14 | 51203 | G | GACA | intergenic variant | KHVJ037-KHVJ038 | 0.01 | 0.99 | 0.89 | 0.11 |
| 15 | 62988 | CT | C | intergenic variant | KHVJ049-KHVJ050 | 0.00 | 1.00 | 0.00 | 1.00 |
| 16 | 83669 | G | C | missense variant | KHVJ056 | 0.74 | 0.26 | 0.01 | 0.99 |
| 17 | 85005 | TG | T | intergenic variant | KHVJ057-KHVJ058 | 0.71 | 0.29 | 0.65 | 0.35 |
| 18 | 104329 | C | T | missense variant | KHVJ070 | 0.87 | 0.13 | 0.73 | 0.27 |
| 19 | 104341 | T | C | missense variant | KHVJ070 | 0.84 | 0.16 | 0.84 | 0.16 |
| 20 | 104472 | T | G | missense variant | KHVJ070 | 0.79 | 0.21 | 0.81 | 0.19 |
| 21 | 105490 | C | T | missense variant | KHVJ070 | 0.68 | 0.32 | 0.73 | 0.27 |
| 22 | 112097 | C | G | missense variant | KHVJ071 | 0.84 | 0.16 | 0.68 | 0.32 |
| 23 | 124125 | CTT | C | intergenic variant | KHVJ075-KHVJ076 | 0.16 | 0.84 | 0.76 | 0.24 |
| 24 | 124140 | GT | G | intergenic variant | KHVJ075-KHVJ076 | 0.14 | 0.86 | 0.10 | 0.90 |
| 25 | 124200 | AGGGGGG | A.AG | intergenic variant | KHVJ075-KHVJ076 | 0.06 | 0.65 | 0.11 | 0.89 |
| 26 | 138300 | C | A | missense variant | KHVJ082 | 0.00 | 1.00 | 0.08 | 0.64 |
| 27 | 151633 | GT | G | intergenic variant | KHVJ090-KHVJ091 | 0.90 | 0.10 | 0.00 | 1.00 |
| 28 | 151649 | T | TA | intergenic variant | KHVJ090-KHVJ091 | 0.90 | 0.10 | 0.89 | 0.11 |

| | | | | | | | | | |
|---|---|---|---|---|---|---|---|---|---|
| 29 | 151780 | G | GTGA | intergenic variant | KHVJ090-KHVJ091 | 0.89 | 0.11 | 0.88 | 0.12 |
| 30 | 152587 | G | A | missense variant | KHVJ091 | 0.84 | 0.16 | 0.87 | 0.13 |
| 31 | 158882 | C | G | missense variant | KHVJ096 | 0.78 | 0.22 | 0.90 | 0.10 |
| 32 | 159828 | G | T | missense variant | KHVJ096 | 0.00 | 1.00 | 0.79 | 0.21 |
| 33 | 187878 | GTCT | G | inframe deletion | KHVJ111 | 0.22 | 0.78 | 0.00 | 1.00 |
| 34 | 205798 | ACTT | A | inframe deletion | KHVJ123 | 0.34 | 0.66 | 0.14 | 0.86 |
| 35 | 216278 | T | TCAACAGCAG | inframe insertion | KHVJ131 | 0.00 | 1.00 | 0.29 | 0.71 |
| 36 | 223325 | A | C | missense variant | KHVJ138 | 0.00 | 1.00 | 0.00 | 1.00 |
| 37 | 226260 | A | C | missense variant | KHVJ140 | 0.00 | 1.00 | 0.00 | 1.00 |
| 38 | 230658 | C | A | missense variant | KHVJ144 | 0.00 | 1.00 | 0.00 | 1.00 |
| 39 | 247692 | G | C | synonymous variant | KHVJ153 | 0.03 | 0.97 | 0.00 | 1.00 |
| 40 | 253848 | C | CGCT | inframe insertion | KHVJ157 | 0.00 | 1.00 | 0.03 | 0.97 |
| 41 | 257090 | C | T | missense variant | KHVJ159 | 0.00 | 1.00 | 0.00 | 1.00 |
| 42 | 258111 | C | T | intergenic variant | KHVJ159-KHVJ160 | 0.00 | 1.00 | 0.00 | 1.00 |
| 43 | 265700 | TTGC | T | inframe insertion | KHVJ165 | 0.87 | 0.13 | 0.00 | 1.00 |
| 44 | 269057 | TTCCTCC | T | inframe insertion | KHVJ166 | 0.00 | 1.00 | 0.84 | 0.16 |

| | | | | | | | | | |
|---|---|---|---|---|---|---|---|---|---|
| 45 | 269368 | T | C | missense variant | KHVJ166 | 0.16 | 0.84 | 0.01 | 0.99 |
| 46 | 270260 | A | AGAGGCAGAG | inframe insertion | KHVJ166/KHVJ167 | 0.06 | 0.94 | 0.15 | 0.85 |
| 47 | 270518 | C | CT | frameshift variant | KHVJ166/KHVJ167 | 0.10 | 0.90 | 0.03 | 0.97 |
| 48 | 270520 | G | GCCCTT | frameshift variant | KHVJ166/KHVJ167 | 0.09 | 0.91 | 0.15 | 0.85 |
| 49 | 270562 | C | T | missense variant | KHVJ166/KHVJ167 | 0.01 | 0.99 | 0.14 | 0.86 |
| 50 | 270749 | C | CACAGACT | intergenic variant | KHVJ167-KHVJ168 | 0.07 | 0.93 | 0.03 | 0.97 |
| 51 | 270789 | A | AGC | intergenic variant | KHVJ167-KHVJ168 | 0.14 | 0.86 | 0.05 | 0.95 |
| 52 | 270792 | CT | C | intergenic variant | KHVJ167-KHVJ168 | 0.14 | 0.86 | 0.14 | 0.86 |
| 53 | 270796 | AGC | A | intergenic variant | KHVJ167-KHVJ168 | 0.12 | 0.88 | 0.14 | 0.86 |
| 54 | 270801 | C | CT | intergenic variant | KHVJ167-KHVJ168 | 0.11 | 0.89 | 0.14 | 0.86 |
| 55 | 270911 | GACAGAGACACAAGACAGAC | G | intergenic variant | KHVJ167-KHVJ168 | 0.00 | 1.00 | 0.16 | 0.84 |
| 56 | 271009 | C | CAA | intergenic variant | KHVJ167-KHVJ168 | 0.00 | 1.00 | 0.00 | 1.00 |
| 57 | 271715 | TTCAGTGTCCTCC | T | inframe deletion | KHVJ168 | 0.68 | 0.32 | 0.00 | 1.00 |
| 58 | 274370 | AATC | A | intergenic variant | KHVJ170-KHVJ171 | 0.25 | 0.75 | 0.67 | 0.33 |

All variations located in the terminal repeats appear only once. Predicted ORFs were deduced from the genome annotation of KHV-J (AP008984). Missense variant indicates a nucleotide substitution that modifies the amino acid; synonymous variant denotes a nucleotide substitution that does not modify the amino acid; frameshift variant indicates an insertion or a deletion that changes the reading frame; intergenic variant points to a mutation located between two predicted open reading frames. Only variations <100 bp were listed.
